# P-1642. Epidemiology of Antimicrobial-Resistant Pathogens Across Eras of SARS-CoV-2 Variants in a Large New Jersey Hospital

**DOI:** 10.1093/ofid/ofaf695.1818

**Published:** 2026-01-11

**Authors:** Ali Ibrahim, Keith S Kaye, Pavan Yalamanchili, Karena Zhao, John P Mills, Michele Pedrani, Thomas Kirn, Ahmed Abdul Azim, Swati kumar, Navaneeth Narayanan

**Affiliations:** Rutgers - New Brunswick, Edison, NewJersey; Rutgers Robert Wood Johnson Medical School, New Brunswick, NJ; Rutgers Robert Wood Johnson Medical School, New Brunswick, NJ; Rutgers University, New Brunswick, New Jersey; Rutgers Robert Wood Johnson Medical School, New Brunswick, NJ; Robert Wood Johnson University Hospital, New Brunswick, New Jersey; Rutgers Robert Wood Johnson Medical School, New Brunswick, NJ; Rutgers Robert Wood Johnson Medical School, New Brunswick, NJ; Rutgers Robert Wood Johnson Medical School, New Brunswick, NJ; Rutgers University Ernest Mario School of Pharmacy & Robert Wood Johnson University Hospital, New Brunswick, NJ

## Abstract

**Background:**

The COVID-19 pandemic placed burden on infection prevention programs in US hospitals. The rapidly evolving viral variants had heterogeneous characteristics (e.g., severity of illness or transmissibility) that may have differentially impacted practices and outcomes. No studies have systematically evaluated the impact of COVID-19 on the incidence of antimicrobial resistant (AR) pathogens during different phases of the pandemic. We analyze the impact of different SARS-CoV-2 variants on the time-varying incidence of AR pathogens.

**Figure:**
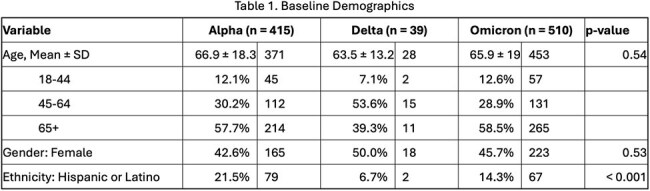

**Figure:**
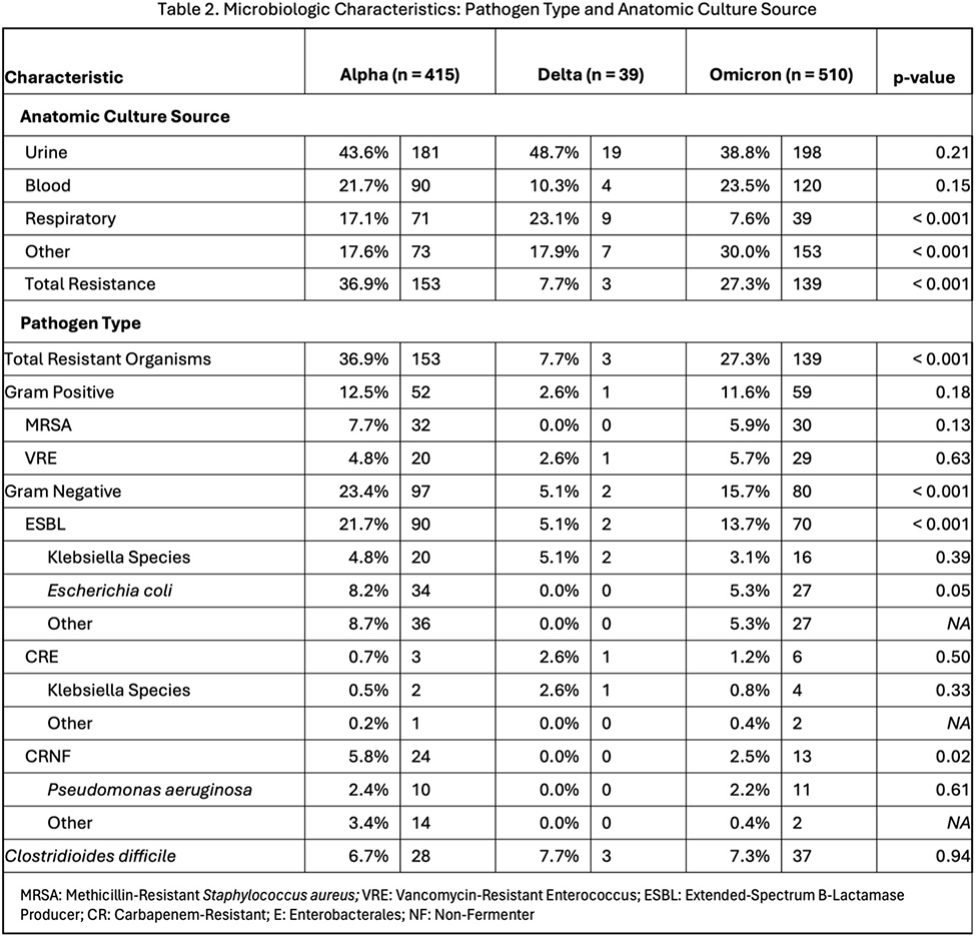

**Methods:**

Data were abstracted from 3/2020-12/2023 to identify cases of co-occurring episodes of COVID-19 infection and AR pathogens from clinical cultures (COVID/AR) including methicillin-resistant *Staphylococcus aureus* (MRSA), vancomycin-resistant Enterococcus (VRE), ceftriaxone-resistant Enterobacterales (ESBL), carbapenem-resistant Enterobacterales (CRE), carbapenem-resistant non-fermenters (CRNF) and *Clostridioides difficile* (CDI). Rates and characteristics of COVID/AR cases were compared during three major variant periods: Alpha (3/2020-6/2021), Delta (6/2021-12/2021) and Omicron (12/2021-12/2023).

**Figure:**
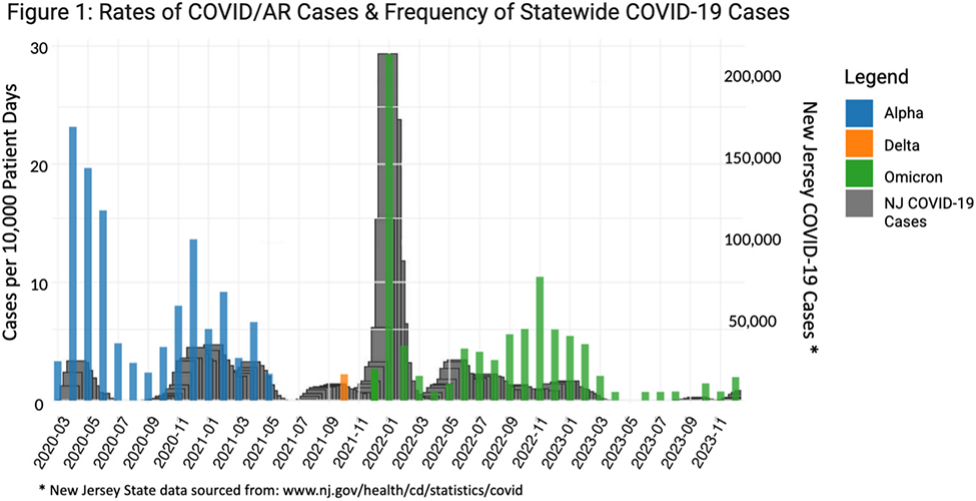

**Figure:**
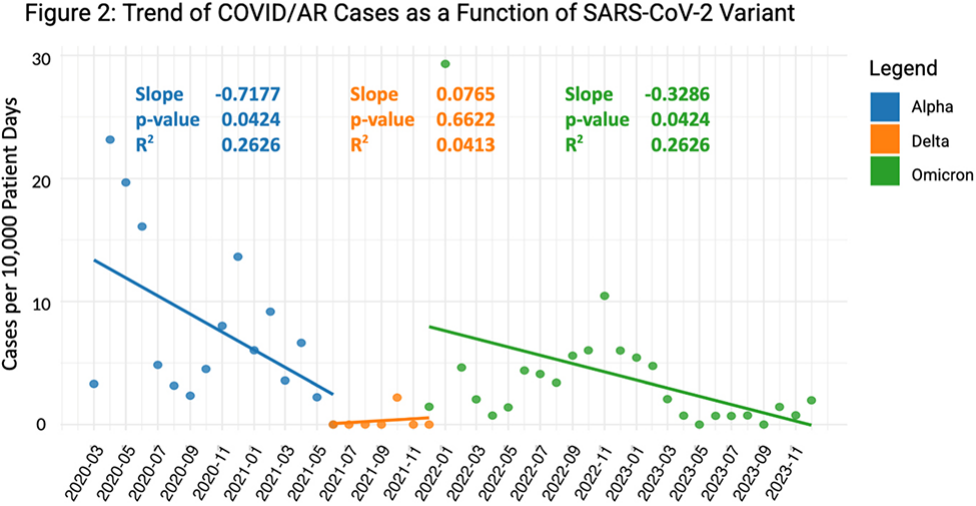

**Results:**

There were 934 COVID/AR episodes occurring among 748 unique patients. Mean age was 66 years, 52% were male, and 15% were Hispanic. The most common pathogen occurring in COVID/AR episodes were CDI (68, 7.1%), MRSA (62, 6.4%), and *E. coli* (61, 6.3%). During the three variant periods, there were no significant differences in patient demographics except for fewer Hispanic patients in Delta period (Table 1). Frequency of AR pathogens (ESBL and CRNF) and anatomic culture source differed by variant period (Table 2). The frequency of COVID/AR episodes mirrored the frequency of statewide COVID-19 cases (Figure 1). There were significant decreases in occurrence of COVID/AR cases during Alpha and Omicron periods, but not during Delta period (Figure 2).

**Conclusion:**

AR pathogen type and source varied as a function of variant type. Rates of COVID/AR decreased substantially during Alpha and Omicron periods but not during Delta period. These differences in COVID/AR incidence trends during the different variant periods might relate to differences in case-mix, transmissibility, and/or infection control processes.

**Disclosures:**

Keith S. Kaye, MD, MPH, AbbVie: Advisor/Consultant|GSK: Advisor/Consultant|Merck: Advisor/Consultant|Shionogi: Advisor/Consultant Thomas Kirn, MD PhD, BD: Advisor/Consultant|BD: Honoraria

